# Localizing Microemboli within the Rodent Brain through Block-Face Imaging and Atlas Registration

**DOI:** 10.1523/ENEURO.0216-21.2021

**Published:** 2021-08-02

**Authors:** Matthew W. McDonald, Matthew S. Jeffers, Melissa Filadelfi, Andrea Vicencio, Gavin Heidenreich, Junzheng Wu, Gergely Silasi

**Affiliations:** Department of Cellular and Molecular Medicine, Faculty of Medicine, University of Ottawa, Ottawa, Ontario, Canada

**Keywords:** block-face, histology, microinfarcts, mouse, stroke, vascular dementia

## Abstract

Brain microinfarcts are prevalent in humans, however because of the inherent difficulty of identifying and localizing individual microinfarcts, brain-wide quantification is impractical. In mice, microinfarcts have been created by surgically introducing microemboli into the brain, but a major limitation of this model is the absence of automated methods to identify and localize individual occlusions. We present a novel and semi-automated workflow to identify the anatomic location of fluorescent emboli (microspheres) within the mouse brain through histologic processing and atlas registration. By incorporating vibratome block-face imaging with the QuickNII brain registration tool, we show that the anatomic location of microspheres can be accurately registered to brain structures within the Allen mouse brain (AMB) atlas (e.*g*., somatomotor areas, hippocampal region, visual areas, etc.). Compared with registering images of slide mounted sections to the AMB atlas, microsphere location was more accurately determined when block-face images were used. As a proof of principle, using this workflow we compared the distribution of microspheres within the brains of mice that were either perfused or immersion fixed. No significant effect of perfusion on total microsphere number or location was detected. In general, microspheres were distributed brain-wide, with the largest density found in the thalamus. In sum, our block-face imaging workflow enables efficient characterization of the widespread distribution of fluorescent microemboli, facilitating future investigation into the impact of microinfarct load and location on brain health.

## Significance Statement

Cerebral microemboli are small materials, such as a blood clot or atherosclerotic plaque, that pass through the circulation until being lodged within downstream vasculature and occasionally resulting in localized cell death (microinfarction). The largest limitation of microemboli models of microinfarction in rodents is the lack of efficient methods for accurately quantifying the brain-wide distribution of occlusions in individual subjects. Compared with traditional histology, incorporating our block-face imaging workflow significantly reduces the time required to register all emboli from a single brain (from days to hours). Going forward, this workflow will allow researchers to more efficiently characterize the global distribution of microemboli in mice and facilitate the stratification of mice based on microsphere load or location.

## Introduction

Brain microinfarcts are found in 30% of individuals over the age of 65 and are associated with stroke, vascular cognitive impairment, and Alzheimer’s disease (Soontornniyomkij et al., 2010; Arvanitakis et al., 2011; Brundel et al., 2012; Summers et al., 2017). Microinfarcts typically do not manifest in acute deficits, cannot be easily visualized with conventional magnetic resonance imaging (MRI), and are most-often not discovered until histologic sampling postmortem (Brundel et al., 2012; Westover et al., 2013). Since the majority of microinfarcts in the human brain go undetected, it is extremely difficult to thoroughly characterize brain-wide microinfarction and determine the impact of microinfarct load (i.e., total number of microinfarcts) or location on functional outcomes.

Microinfarction has been modelled in rodents by selectively targeting penetrating arterioles with laser-induced damage (Shih et al., 2013; Taylor et al., 2016; Summers et al., 2017; Lubart et al., 2018), bilateral carotid artery stenosis (Holland et al., 2015), or surgically injecting microemboli into the brain circulation (Rapp et al., 2008; Lam et al., 2010; Silasi et al., 2015). The injection of fluorescent microspheres represents one of the most promising models of microinfarction (Silasi et al., 2015), as each occlusion site can be visualized through fluorescence microscopy and the distribution of emboli mimic the brain-wide distribution of microinfarction in humans (Westover et al., 2013). However, similar to the clinical difficulty in globally characterizing all microinfarcts, a significant limitation of emboli models is the absence of automated methods for readily localizing each occlusion site within the rodent brain. Indeed, manually determining the location of microemboli across the entire brain is labor-intensive and prone to human error. It requires serially sectioning the entire brain, incorporating an immunolabel or stain to visualize brain structures, imaging each brain section, and manually allocating each microsphere to brain regions by referencing a species-appropriate atlas, such as the Allen mouse brain (AMB; Lein et al., 2007; Wang et al., 2020). Because of these impediments, only a few studies have attempted to characterize the location and distribution of fluorescent microspheres postinjection, and those that have, used small subsets of mice (Silasi et al., 2015; Balbi et al., 2019). In order to further refine preclinical models of microinfarction using microemboli, standardized and preferably automated methods to readily quantify the location of microemboli are needed.

A number of methods have been developed to register serial histologic images to three-dimensional AMB atlas space, including but not limited to the QuickNII tool (Puchades et al., 2019), Atlas Fitter (Kopec et al., 2011), aMAP (Niedworok et al., 2016), and AMaSiNe (Song et al., 2020). QuickNII has a number of advantages compared with other atlas registration techniques as it requires minimal user guided transformations, does not introduce distortions to the original experimental images, and it can be used by those without programming knowledge. When registering histologic images to an atlas, the tissue deformation and related artifacts induced by mounting sections on slides reduces the accuracy of atlas registration. Therefore, a number of imaging approaches to facilitate 3D whole-brain microscopy have also been developed including light-sheet microscopy and serial section two-photon microscopy in cleared tissue (Aswendt et al., 2017). However, these advanced forms of microscopy require specialized equipment and expertise that are not available in most laboratories, therefore we developed a block-face imaging method that can be incorporated with minimal expertise and hardware modifications. Block-face imaging is a technique that captures serial images of the tissue block during sectioning; reducing the time it takes to digitize brain sections, limiting tissue manipulation, and maintaining the natural shape of the tissue. Capturing serial images of fluorescent microspheres during sectioning and incorporating a brain registration tool, such as the QuickNII, could provide high-throughput and accurate neuroanatomical localization of microemboli.

We developed and validated a novel and semi-automated workflow to localize fluorescent microspheres within the rodent brain through block-face imaging and atlas registration. The accuracy of registering microspheres to the AMB atlas from block-face images was also compared with images collected from slide-mounted sections. As proof of principle for our workflow, and to further characterize the fluorescent microsphere model, we determined how microspheres distribute across the entire mouse brain. In addition, we assessed whether perfusion at the time of killing alters the number, as well as the distribution of microspheres within the brain.

## Materials and Methods

All animal procedures were performed in accordance with the University of Ottawa’s animal care committee’s regulations. In this two-part experiment, a total of 35 male Thy1-ChR2-YFP mice (B6.Cg-Tg(Thy1-COP4/EYFP)18Gfng/J; 007612; The Jackson Laboratory) were used (2.5–5 months of age). First, we validated our block-face imaging workflow in a subset of mice (*n* = 5; referred to as the validation experiment). Subsequently, the remaining mice were allocated to our proof of principle experiment to examine microsphere distribution and assess the effect of perfusion on the distribution of microspheres (*n* = 30; referred to as proof of principle experiment).

### Microsphere injection

Under isoflurane anesthesia (4% induction, 1.5% maintenance) the common carotid artery (CCA) was isolated through a neck incision, the external carotid artery was transiently clamped, and 2000 fluorescent microspheres (20 μm in diameter, Polysciences, Fluoresbrite YG catalog #19096-2) were injected through the CCA with a 33-G needle (100-μl solution, diluted in saline). Consistent with the diameter of penetrating arterioles (Hall et al., 2014), microspheres typically occlude vessels with a diameter ∼11 μm (Silasi et al., 2015). Immediately after the needle was removed, bioabsorbable Gelfoam (Pfizer) was applied to the CCA to control bleeding. The neck incision was closed after 5 min to allow for sufficient blood clotting at the needle injection site. In our validation experiment, 21 d following the injection, mice were anesthetized with euthanyl and perfused (3.6 ml/min) transcardially with phosphate buffered saline (pH 7.4) followed by 4% paraformaldehyde. Brains remained in 4% paraformaldehyde until sectioning. Mice in the proof of principle experiment were killed 24 h after microsphere injection.

### Preparing tissue for sectioning

**Figure 1.**
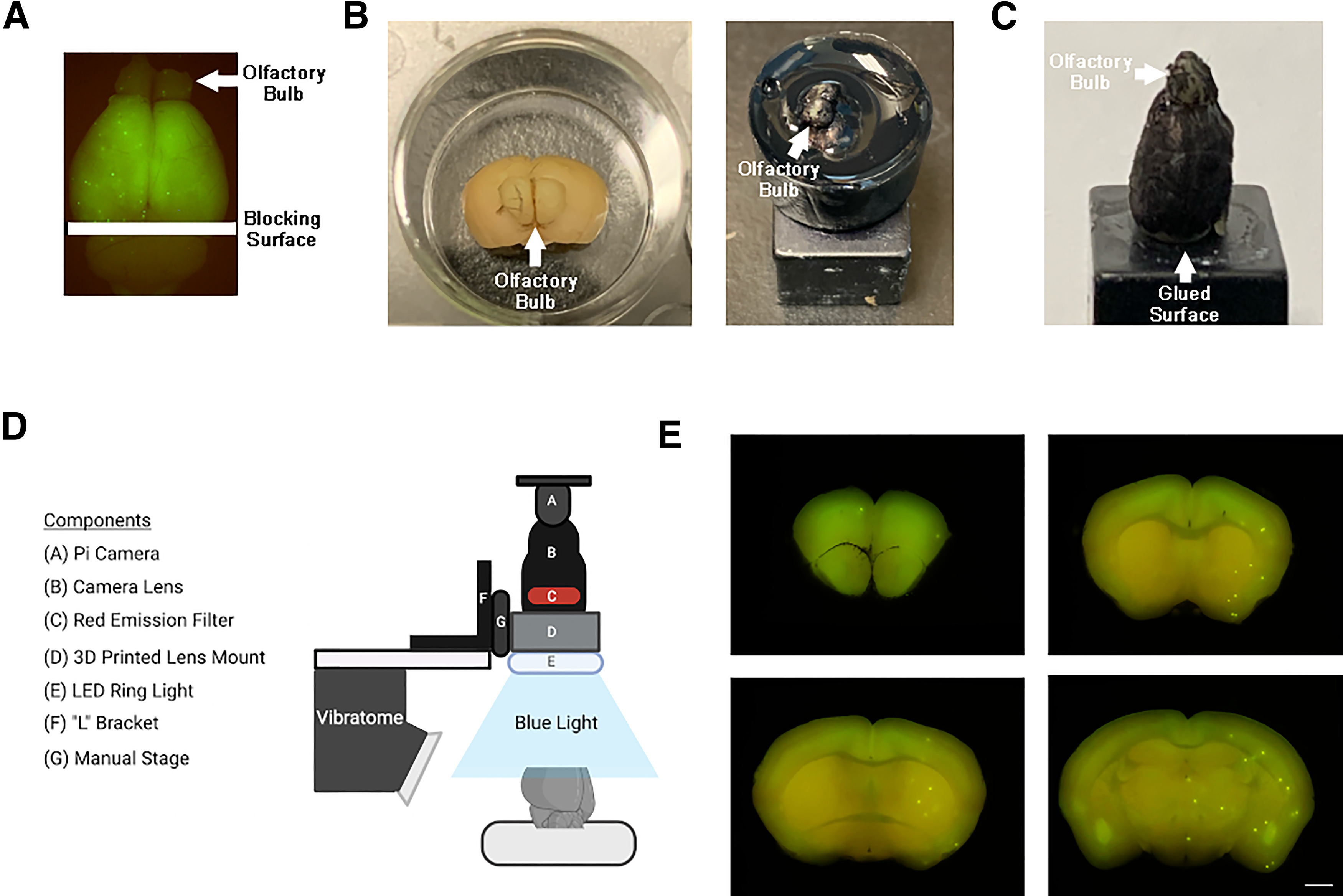
Block-face imaging setup. ***A***, Brains were blocked coronally at the level of the colliculus. ***B***, To ensure a black background during block-face imaging, brains were embedded in 2% agarose darkened with India Ink, using a 45-well plate as a mold. Once solidified, the block was extracted from the well plate and glued to the vibratome chuck for sectioning. ***C***, Alternatively, the brain can be directly glued to the vibratome chuck and black ink can be applied directly to the brain surface. ***D***, Visual representation of the block-face imaging setup. ***E***, Representative block-face images of mouse brain with fluorescent microspheres (scale bar: 1 mm).

Brains were blocked coronally with a razor blade at the level of the colliculus (Fig. 1*A*). Using a 45-well plate (Fig. 1*B*, left), brains were embedded in a 2% agarose matrix dyed with India Ink (Speedball Super Black Waterproof India Ink). Once the agarose gel dried, the agarose block was easily removed from the well and glued (LePage Super Glue) to the vibratome chuck (Fig. 1*B*, right). In the proof of principle experiment, rather than using a tissue embedding medium, black marker (LePen alcohol-based permanent ink) was applied to the brain surface (Fig. 1*C*). Both methods ensure a black background throughout the block-face imaging procedure. The main advantage of applying ink directly to the surface of the brain is that it reduces tissue manipulation during sectioning, as agarose embedded sections need to be separated from the surrounding agarose before placing on a slide.

### Brain sectioning

Block-face imaging was performed with a Raspberry Pi Camera (8MP, Camera Board V2) equipped with a varifocal (6–22 mm) CCTV lens (Binchil, model 149129). An orange gelatin filter was cut to fit on the back aperture of the lens, and a blue LED ring light (Adafruit; Flora NeoPixel Ring, P1463) was mounted below the lens on a custom 3D-printed camera mount (Fig. 1*D*; see Extended Data 1). Images were acquired (2592 × 1944 pixels; 6.5 μm/pixel) on a Raspberry Pi 3 microcomputer (Raspberry Pi Foundation) using a custom Python script (see Extended Data 1). The entire brain was serially sectioned on a vibratome (Vibratome Series 1500) at 100 μm and coronal block-face images were acquired before each section (Fig. 1*E*). Therefore, the *z*-plane resolution of our microsphere localization is equal to the thickness of our vibratome sections (100 μm). To reduce ambient room light, images were collected in the dark. Brain sections were collected from the water bath with a brush and mounted on positively-charged gelatin coated slides, stained with DAPI (ThermoFisher Scientific), and imaged using a Zeiss Axio Imager M2 microscope at 2.5× magnification.

### Image processing

**Figure 2.**
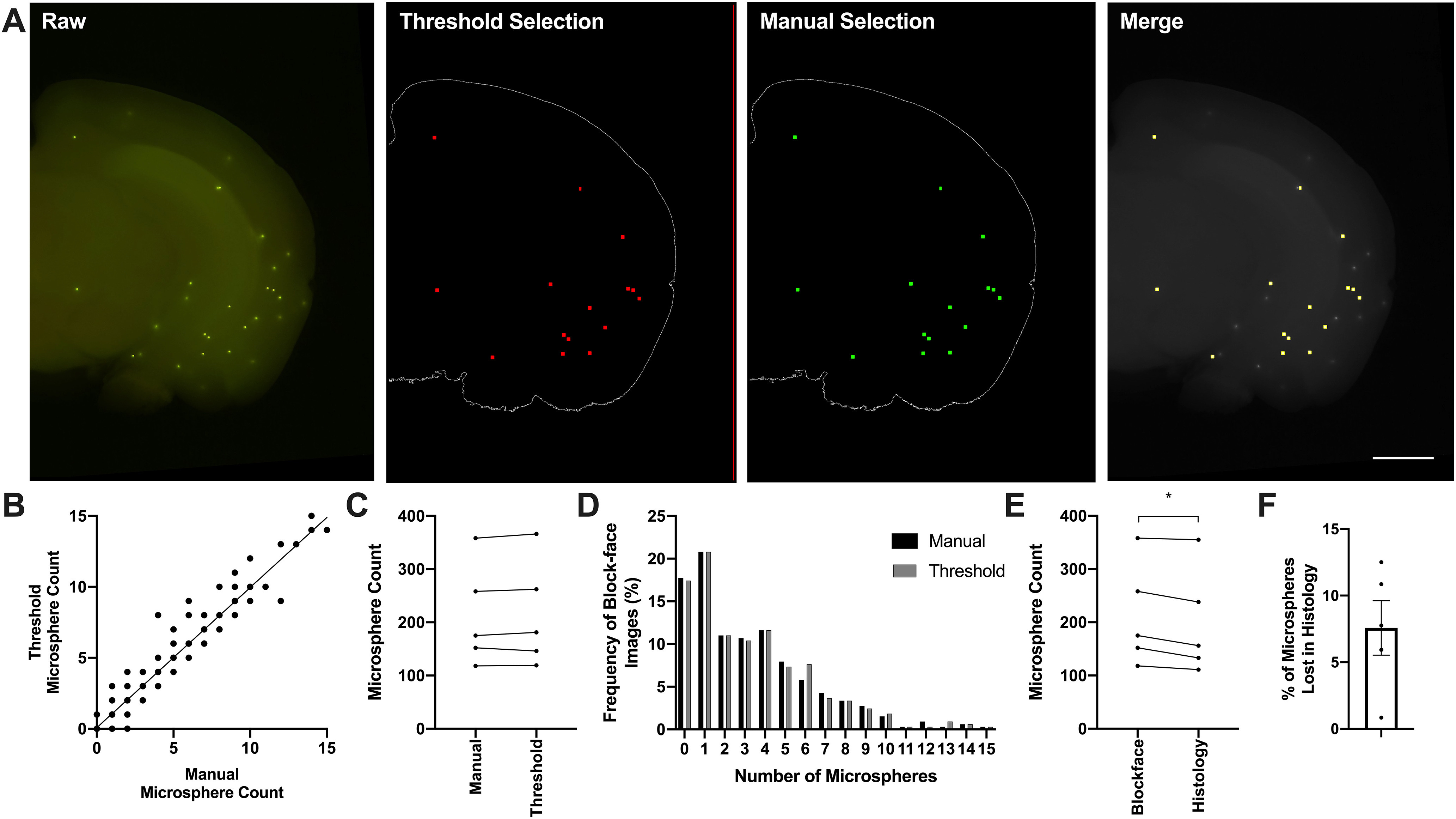
Microsphere detection. ***A***, Validation of image thresholding to produce binary segmentation masks of individual microspheres. Left, Block-face image with visible fluorescent microspheres (green). Fluorescent microspheres are visible ∼300–400 μm into the tissue block. Following image thresholding to distinguish microspheres, serial images are subtracted from each other to determine the microsphere’s true location. Middle left, Microspheres (red) detected with image threshold after subtraction of serial images. Middle right, Microspheres manually counted by an experimenter after subtraction of serial images (green). Right, Overlay of raw block-face image with microspheres manually selected and thresholded (yellow, agreement between manual and image threshold); scale bar: 1 mm. ***B***, Correlation between microspheres identified by experimenter and microspheres that were detected from thresholded image. Each data point represents the number of microspheres identified manually or detected from thresholded image of each coronal image (*N* = 327; *n* = 64–67 per brain). ***C***, Total number of microspheres manually identified by an experimenter or from thresholded image for each brain. ***D***, The number of microspheres identified by an experimenter or thresholded image in each block-face image (*N* = 327). ***E***, Number of microspheres manually counted from block-face images compared with the number of microspheres manually counted in histologic sections. ***F***, Percent of microspheres lost in histologic sections when compared with the equivalent block-face image. Data are mean ± SD. Asterisk = significance based on paired *t*-test (*p* < 0.05).

Serial block-face images were processed using a custom ImageJ/Fiji macro script (see Extended Data 2). Serial images were rotated based on the user drawing a line down the midline of a single brain image followed by the images being automatically aligned using the “StackReg” plugin (https://imagej.net/StackReg) using the “Rigid Body” transformation. Microspheres were detected within each coronal image by creating a binary segmentation mask based on the intensity of the microsphere signal in the green color channel of the eight-bit image. Specifically, pixels with an intensity value between 0–140 were excluded and classified as background signal, while pixels with an intensity from 140 to 256 were classified as microsphere signal. Because of the highly fluorescent nature of the microspheres, they are visible at depths of up to 400 μm from the surface, meaning that not all visible microspheres in a given image are located with the first 100-μm section that is removed from the tissue block (Fig. 2*A*, left). To account for cases where the same microsphere was visible in sequential block-face images, adjacent binary segmentation masks were subtracted from each other; therefore, the last image that a microsphere was detected in was determined to be its true *z*-plane position (Fig. 2*A*, middle left). For manual identification, an experimenter blinded to the automated segmentation of microspheres was instructed to manually identify every visible microsphere using the point tool in ImageJ, and a binary segmentation mask was produced based on the identified *x*/*y* coordinate. Subsequently, each segmented pixel was dilated to a 20-μm diameter to match the size of a microsphere. Lastly, to determine the *z*-plane where the microsphere was located a final segmentation mask was created using the same image subtraction as described above (Fig. 2*A*, middle right). To validate our image-intensity based method of automated microsphere detection we compared the number of microspheres detected from thresholded images versus manually selected microspheres (Fig. 2*A*, right). Manual segmentation masks were only used to validate the microsphere thresholding parameters.

### Alignment to AMB atlas

**Figure 3.**
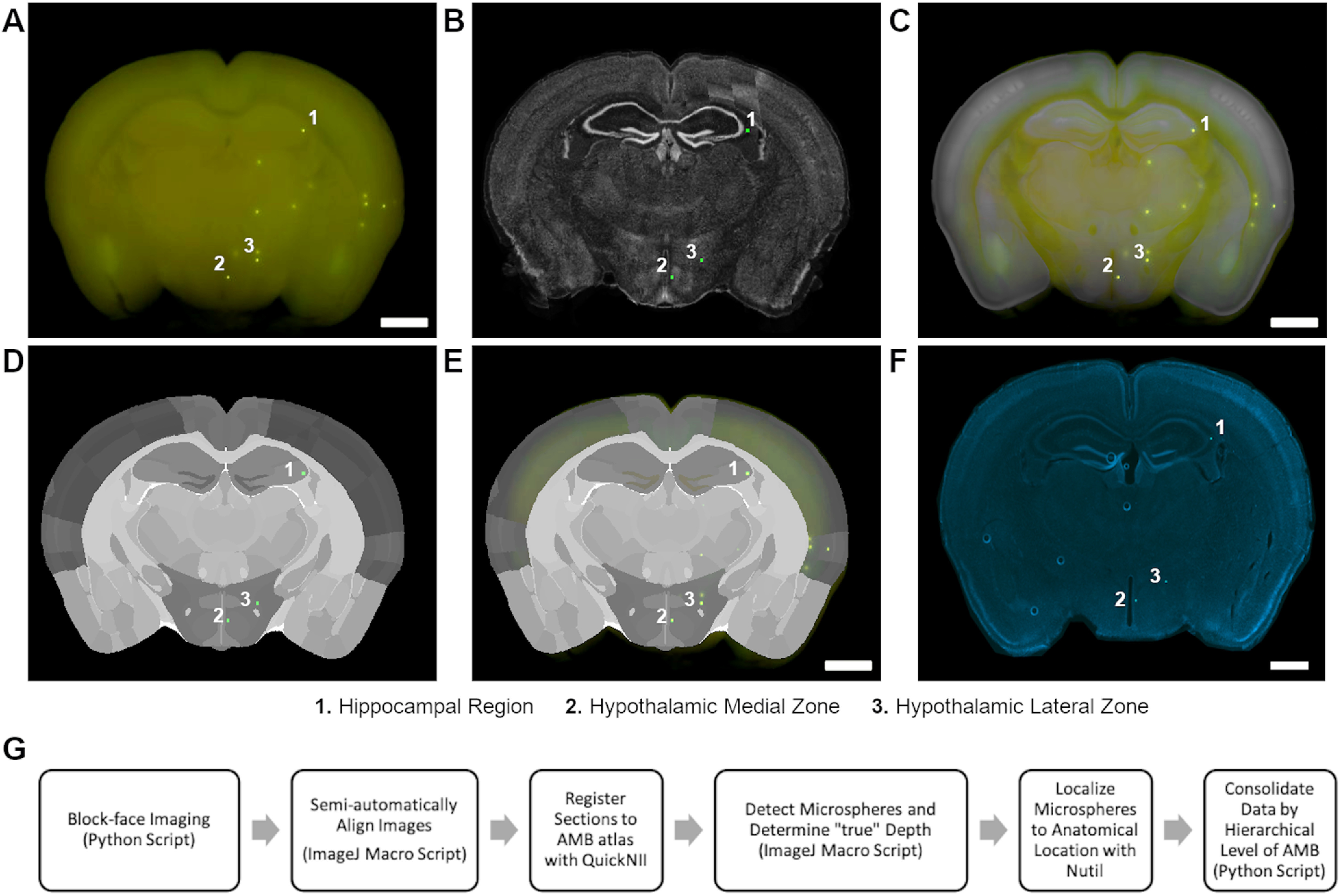
Block-face imaging workflow. ***A***, Block-face image with three surface microspheres identified. ***B***, Microspheres detected from block-face image registered to AMB Nissl image using QuickNII. Microspheres detected with image threshold (green). ***C***, Block-face image “anchored” to Nissl image of AMB atlas. ***D***, AMB atlas segmentation map of block-face image with overlay of microspheres detected with image threshold (green). Each shade of gray is a unique brain region within the AMB (2017). ***E***, Block-face image anchored to AMB atlas segmentation map. ***F***, Slide-mounted brain section collected after acquiring block-face image. DAPI (blue) and microspheres (green). ***G***, Visual representation of the steps of our block-face imaging workflow and atlas registration of microspheres. For example, using this workflow and Nutil, three surface microspheres were identified to be in the (1) hippocampal region, (2) medial hypothalamic zone, and (3) the lateral hypothalamic zone (scale bar: 1 mm).

Using the QuickNII tool, five 2D block-face images were localized to AMB atlas space (2017) by “anchoring” to visible anatomic structures (Puchades et al., 2019). Anchoring is the process of superimposing customized 2D AMB atlas images constructed from QuickNII onto block-face images (Fig. 3*A–E*). Based on visual landmarks in the block-face image (e.g., cortical boundaries, white matter tracts, hippocampus, etc.), the user identified the correct location of the block-face image in AMB atlas space. The user amended the size of the atlas image and adjusted mediolateral (or dorsoventral) angles as necessary (see hippocampal region in Fig. 3*B*). To reduce the number of user-inputted transformations in QuickNII, the position and angles identified during anchoring were propagated throughout the block-face image series.

To localize serial block-face images to the appropriate anterior-posterior (A-P) coordinates of AMB atlas space, coronal images were first anchored to (1) where the corpus callosum first crosses the midline (Fig. 1*E*, top right) and (2) where the anterior commissure first crosses the midline (Fig. 1*E*, bottom left). Based on these two identified A-P coordinates, and the number of images between these two anchored images, QuickNII established the A-P location of the remaining serial block-face images. Spatial registration was further refined by anchoring to coronal images from (3) the anterior of the brain containing frontal cortex and olfactory areas (Fig. 1*E*, top left) and from (4) the anterior and (5) posterior hippocampus (Fig. 1*E*, bottom right). After completing these anchoring steps, QuickNII has a built-in function to visually identify that transformations are accurate across images. If further validation is necessary, each block-face image can also be superimposed on to Nissl images or atlas segmentation maps to verify successful anchoring (Fig. 3*A–E*).

After anchoring sections with QuickNII, binary segmentation masks were automatically generated (i.e., thresholded microsphere image after serial image subtraction) using our custom ImageJ script (see Extended Data 2). Using this script, the user also had the option to semi-automatically define each hemisphere by delineating the midline of block-face images (Fig. 2, middle images). This step is necessary for determining which hemisphere (right vs left) the microsphere is located in. Subsequently, the precise location of each microsphere was determined by using the complementary software Nutil (Groeneboom et al., 2020). Nutil utilizes the AMB spatial coordinates of the block-face images obtained from QuickNII and combines it with the binary segmentation masks of your objects of interest (i.e., consolidated thresholded microsphere image) to localize objects (i.e., microspheres) to known anatomic structures based on the AMB atlas (Fig. 3*A–E*). The workflow is shown visually in Figure 3*G*.

### Workflow validation

First, we compared the ability of our block-face imaging workflow to allocate all microspheres from our validation subset (five brains; *N* = 1061 total microspheres) to brain regions easily identifiable in block-face images by a blinded experimenter (olfactory areas, isocortex, corpus callosum, hippocampal region, striatum, thalamus and midbrain, and all other regions). Next, the ability of our block-face imaging workflow to accurately localize microspheres was compared with the traditional method of manual localization of microspheres from histology and referencing the AMB atlas. In our validation subset of brains, we sampled every 20th microsphere identified using our workflow and a blinded experimenter determined its location by referencing the AMB atlas. In addition, using QuickNII we were interested in determining how registration of microsphere location from block-face images compares to registration of microspheres from serial histologic sections, as QuickNII was originally intended. Therefore, microspheres on collected histologic sections were also manually selected by an experimenter to produce a binary segmentation mask, and histologic sections were processed through the workflow. We provide a Python script to facilitate the consolidation of all Nutil outputs into a single data file separating microsphere location by anatomic level of the AMB (see Extended Data 3).

### Proof of principle experiment

Thirty Thy1-ChR2-YFP male mice underwent a microsphere injection, as described above. Mice were killed 1 d following microsphere injection and randomly divided into groups that either underwent transcardial perfusion (3.6 ml/min) with phosphate buffered saline (pH 7.4) and 4% paraformaldehyde or immersion fixation of the brain in 4% paraformaldehyde without perfusion (*n* = 20) for at least 3 d (*n* = 15/group). The mortality rate was 3.3% following the surgery (*n* = 1; day 1, no perfusion group).

Microsphere number and location were determined using our block-face imaging workflow. To calculate total microsphere density per brain, brain volume was determined by summing the area of coronal block-face images and multiplying by section thickness. The anatomic distribution of microspheres in the brain was also compared between experimental groups. The Allen reference atlas ontology is a hierarchical tree, with the first level (“root”) being divided into gray matter (including cell groups and regions), fiber tracts, and ventricular systems (i.e., level 2). From this point onwards, each of these larger regions are further divided into smaller subregions (up to level 12), each with different numbers of “leaves” (Wang et al., 2020). Therefore, every subregion does not always end at the same level of the hierarchal tree and similar structures are not always on the same hierarchical level (Wang et al., 2020). For example, the hierarchical tree of primary motor area, layer 5 would be 9 levels: root → basic cell groups and regions → cerebrum → cerebral cortex → cortical plate → isocortex → somatomotor area → primary motor area → primary motor area, layer 5. Since the distribution of microspheres is widespread, the highest levels of the Allen reference atlas often have limited numbers of microspheres located in them (1 or 2). Therefore, to reduce the number of statistical factors to compare and to decide at which level of the tree we would perform our analysis (i.e., terminal branch), in the no perfusion group we set a criterion that brain regions had to have at least five microspheres to consider moving to the next anatomic subdivision (i.e., higher level). This criterion identified 26 target brain regions. To determine microsphere density in our targeted brain regions, the number of microspheres in a given region were divided by the volume of each structure defined by the AMB atlas (2017).

### Code accessibility

The code/software described in the paper is freely available online (https://github.com/silasilab/microspheres) and available as Extended Data 1, 2 and 3.

10.1523/ENEURO.0216-21.2021.ed1 Extended Data 1 Detailed description of the block-face imaging workflow and Python script for image acquisition. Download Extended Data 1, ZIP file.

10.1523/ENEURO.0216-21.2021.ed2 Extended Data 2 ImageJ script for image processing. Download Extended Data 2, ZIP file.

10.1523/ENEURO.0216-21.2021.ed3 Extended Data 3 Python script to facilitate compiling all microsphere location data. Download Extended Data 3, ZIP file.

### Data analysis

The difference between the number of experimenter-identified and automated thresholding script-identified microspheres was compared using a Pearson correlation and paired *t* test. Similarly, the number of microspheres remaining on histologic sections was compared with the number of microspheres detected from block-face images using a paired *t* test. Total microsphere number, proportion of microspheres, and density of microspheres in a given terminal branch of the anatomic tree in the perfusion experiment were compared using independent samples *t* tests. A significance level of *p* < 0.05 was deemed a statistical difference.

## Results

### Validation of microsphere identification

The accuracy of identifying the microspheres from image thresholding was validated by comparing microsphere counts to those identified manually by an experimenter (Fig. 2). The number of microspheres manually counted by an experimenter was significantly correlated with microspheres detected from thresholded images (*r* = 0.98; *r*^2^ = 0.96; *p* < 0.0001; Fig. 2*B*). The total number of experimenter-identified microspheres throughout the entire brain was not significantly different from the total number determined from thresholded images (*t*_(4)_ = 1.065, *p* = 0.35; Fig. 2*A*, right, *C*). Similarly, the number of microspheres identified by an experimenter in each coronal image was also similar to thresholded images (*t*_(326)_ = 1.087, *p* = 0.28; total number of coronal images = 327; *n* = 64–67 coronal images per brain; Fig. 2*D*). The number of microspheres located on histologic sections were reduced compared with block-face images (*t*_(4)_ = 3.807, *p* = 0.019; Fig. 2*E*), where ∼7.5% of microspheres were lost during sectioning (Fig. 2*F*).

### Validation of microsphere localization

Next, we compared the ability of our block-face imaging workflow to allocate all microspheres to gross anatomic brain regions that are easily identifiable in block-face images by an experimenter (olfactory areas, isocortex, corpus callosum, hippocampal region, striatum, thalamus and midbrain, and all other regions; Table 1). When allocating all microspheres to visible brain regions from block-face images, the level of agreement between our block-face workflow and an experimenter was 93.9% (Table 1). The majority of microspheres were determined to be in the isocortex (38%), followed by the thalamus and midbrain region (25.4%); however, these values were not normalized by volume.

**Table 1:** Agreement between manual allocation of individual microspheres from block-face images and our block-face imaging workflow

		Brain region						
Category	Total counts	Olfactory aeas	Isocortex	Corpus callosum	Hippocampal region	Striatum	Thalamus and midbrain	Other
Agreement	995 (93.9%)	68 (88.3%)	383 (94.6%)	33 (75%)	129 (96.3%	96 (92.3%)	261 (97%)	25 (92.6%)
Disagreement	65 (6.1%)	9 (11.7%)	22 (4.4%)	11 (25%)	5 (3.7%)	8 (7.5%)	8 (3%)	2 (7.4%)
Total	1061	77 (7.3%)	405 (38.2%)	44 (4.2%)	134 (12.6%)	104 (9.8%)	269 (25.4%)	27 (2.5%)

Agreement represents the same allocation between our workflow and manual rating. Overall, there is 93.9% agreement between our workflow and a human rater (*N* = 1061). Microspheres were predominantly found in isocortex and thalamus/midbrain. Data are presented as number of microspheres found in a given region, with the percentage relative to the total counts column found in parenthesis.

Given the high level of agreement between our workflow and manual allocation from an experimenter (∼95%), we proceeded to determine how well our block-face imaging workflow compared with the traditional method of having an experimenter manually localize microspheres from histology. If a microsphere could not be localized in a histologic section (*n* = 5), the next sequential microsphere was used for validation. The percentage of agreement between experimenter allocation and our workflow was determined for each hierarchal level of the AMB (levels 1–10). Since very few anatomic locations are located at levels 11 and 12, we determined level of agreement to level 10. Depending on the hierarchal level of the AMB, the level of agreement between an experimenter and our block-face imaging workflow ranged between 86.5−98.1% (*n* = 52 microspheres sampled; Table 2, upper). With a level of agreement of 94.2%, we determined the highest anatomic level of the AMB that we were confident in localizing microspheres (∼95%) was level 7 (e.g., microspheres in all areas → basic cell groups and regions → cerebrum→ cerebral cortex → cortical plate → isocortex → somatomotor areas). However, the workflow was still accurate to the highest levels (86.5% agreement at level 10) of the anatomic tree. In contrast to our block-face imaging workflow, registering histologic sections (Fig. 3*F*) with QuickNII resulted in less accuracy, with 88.5% agreement at level 7 (Table 2, lower). Similarly, agreement ranged between 80.7% and 98.1% depending on the anatomic level of the AMB.

**Table 2:** At each hierarchal level (Lvl) of the AMB, the agreement between a blinded rater allocating microspheres from histologic images and our block-face imaging workflow (upper) or compared with registering histologic sections with QuickNII (lower)

	Category	Lvl 10	Lvl 9	Lvl 8	Lvl 7	Lvl 6	Lvl 5	Lvl 3/4	Lvl 1/2
Manual histology compared with block-face imaging workflow	Agreement	18 (34.6%)	29 (55.8%)	39 (75%)	45 (86.5%)	50 (96.2%)	50 (96.2%)	50 (96.2%)	51 (98.1%)
	Microsphere not at this level	27 (51.9%)	16 (30.8%)	8 (15.4%)	4 (7.7%)	0 (0%)	0 (0%)	0 (0%)	0 (0%)
	Sum of agreement	45 (86.5%)	45 (86.5%)	47 (90.4%)	49 (94.2%)	50 (96.2%)	50 (96.2%)	50 (96.2%)	51 (98.1%)
	Disagreement	7 (13.5%)	7 (13.5%)	5 (9.6%)	3 (5.8%)	2 (3.8%)	2 (3.8%)	2 (3.8%)	1 (1.9%)
	Total	52	52	52	52	52	52	52	52
Manual histology compared with registering Histologic sections with QuickNII	Agreement	22 (42.3%)	30 (57.7%)	40 (76.9%)	45 (86.5%)	48 (92.3%)	49 (94.2%)	50 (96.2%)	51 (98.1%)
	Microsphere not at this level	20 (38.5%)	14 (26.9%)	4 (7.7%)	1 (1.9%)	0 (0%)	0 (0%)	0 (0%)	0 (0%)
	Sum of agreement	42 (80.7%)	44 (84.6%)	44 (84.6%)	46 (88.5%)	48 (92.3%)	49 (94.2%)	50 (96.2%)	51 (98.1%)
	Disagreement	10 (19.2%)	8 (15.4%)	8 (15.4%)	6 (11.5%)	4 (7.7%)	3 (5.8%)	2 (3.8%)	1 (1.9%)
	Total	52	52	52	52	52	52	52	52

Agreement represents the number of microsphere given the same allocation between the rater and either workflow. Microsphere not at this level, represents the number of microspheres not located at a given level of the hierarchal tree. Some microspheres are not located at specific levels of the hierarchal tree, since every subregion of the AMB hierarchal tree does not end at the same level. Sum of agreement, the number of microspheres correctly allocated up to the specific hierarchal level. Disagreement, represents the number of microspheres incorrectly allocated at the given level. With 94.2% agreement, we are confident we can allocate microspheres from block-face imaging to level 7 of the hierarchal tree of the AMB atlas (e.g., somatomotor, somatosensory, visual, piriform areas, etc.). In contrast, registering histologic sections with QuickNII, rather than block-face images, results in 88.5% agreement at level 7.

### Microsphere number and location

**Figure 4.**
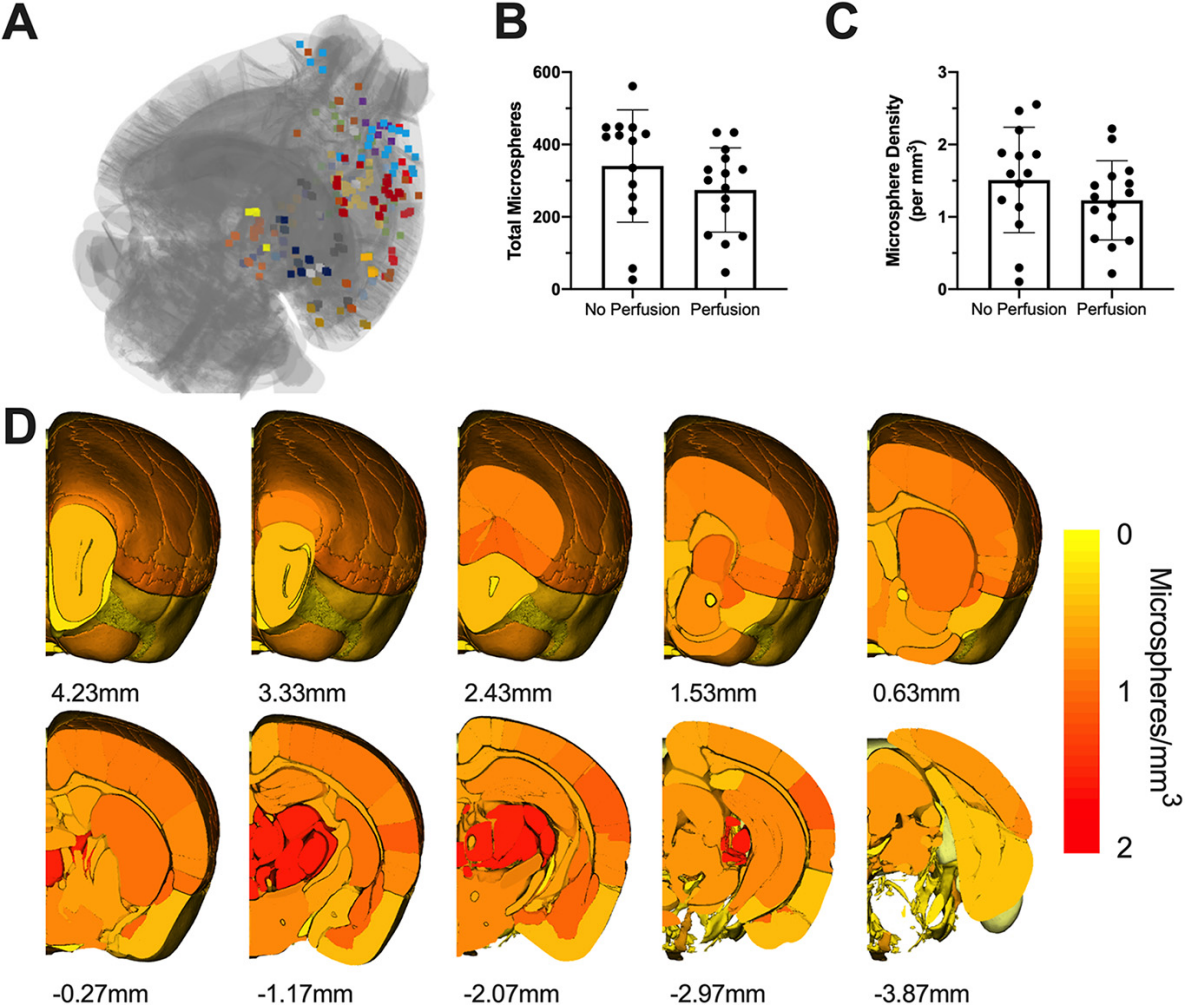
***A***, Representative image of the widespread distribution of microspheres. Data represent the mouse with the mean number of microspheres (301). Each microsphere color represents target brain regions. ***B***, Total number of microspheres in the brain. ***C***, Microsphere density within the total brain volume sampled in each mouse. ***D***, Heat map representing microsphere density (per mm^3^) in target brain regions across all mice (*n* = 29). The largest density of microspheres was found in the thalamus. The A-P coordinate relative to the anterior commissure is provided below each atlas image. Data are mean ± SD.

**Figure 5.**
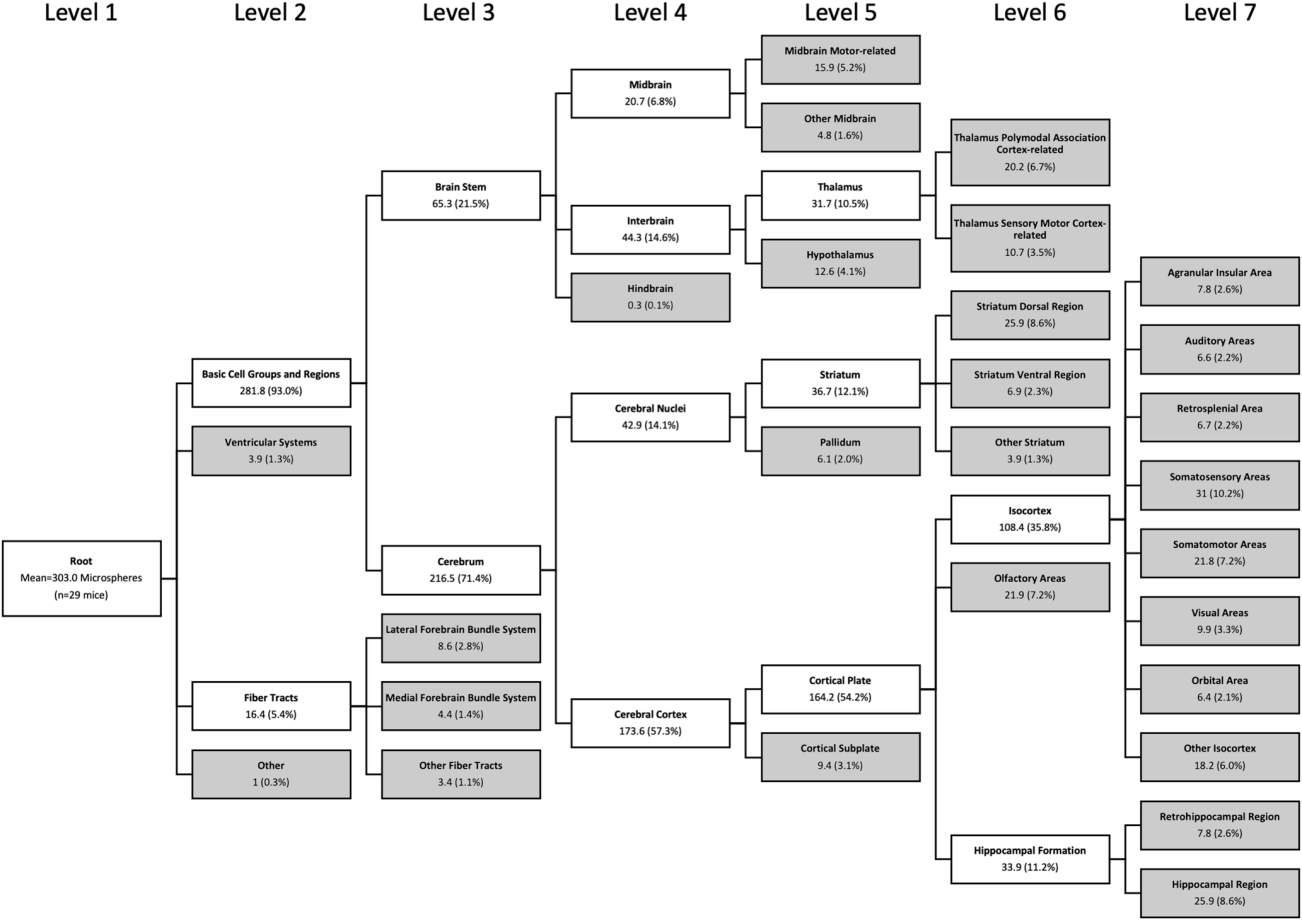
Microsphere location until level 7 AMB hierarchical tree across all animals (*n* = 29). Data are means of the number of microspheres in each anatomic tree location and the proportion relative to total microspheres (in parentheses). Target brain regions (i.e., terminal branches) are in gray.

Microsphere injections resulted in widespread distribution of microspheres (Fig. 4*A*). Although microspheres were injected unilaterally, microspheres were also found in the anterior contralateral hemisphere. No significant effect of perfusion was evident for total microsphere number (*t*_(27)_ = 1.308, *p* = 0.20; Fig. 4*B*) or microsphere density (*t*_(27)_ = 1.177, *p* = 0.25; Fig. 4*C*). When comparing the location of microspheres between experimental groups in the 26 identified brain regions, we found no statistical differences in microsphere number, proportion, or density between groups (*p* > 0.05; Table 3). When all mice were collapsed into a single group, the largest density of microspheres was found in the thalamus (∼1.6 microspheres/mm^3^; Fig. 4*D*; Table 3). For detailed statistics comparing the distribution of microspheres across identified brain regions, see Extended Data 4. The hierarchal tree of microsphere location based on the AMB atlas hierarchy (up to level 7) across all mice is shown in Figure 5.

**Table 3:** Number, proportion, and density of microspheres located in targeted brain regions

	Microsphere number (#)			Microsphere proportion (%)			Microsphere density (#/mm^3^)		
Atlas region of interest	No perfusion	Perfusion	*p* value	No perfusion	Perfusion	*p* value	No perfusion	Perfusion	*p* value
Ventricular systems	4.5 (2.9)	3.3 (1.7)	0.195	1.2 (0.9)	1.5 (1.0)	0.487	0.75 (0.49)	0.56 (0.29)	0.195
Lateral forebrain bundle system	8.4 (5.8)	8.8 (3.2)	0.804	2.7 (1.4)	3.7 (1.6)	0.085	0.60 (0.42)	0.63 (0.23)	0.804
Medial forebrain bundle system	4.2 (3.1)	4.5 (3.3)	0.789	1.2 (0.7)	1.5 (0.9)	0.228	0.60 (0.44)	0.64 (0.46)	0.789
Other fiber tracts	3.5 (2.6)	3.3 (2.2)	0.852	1.1 (1.0)	1.4 (1.0)	0.439	0.24 (0.18)	0.23 (0.15)	0.852
Midbrain, motor-related areas	18.2 (11.7)	13.7 (11.8)	0.315	4.8 (2.3)	4.5 (2.7)	0.719	0.86 (0.55)	0.64 (0.56)	0.315
Other midbrain areas	5.3 (4.6)	4.3 (4.4)	0.576	1.5 (1.1)	1.4 (1.1)	0.820	0.61 (0.53)	0.50 (0.51)	0.576
Hypothalamus	12.9 (7.2)	12.2 (7.7)	0.795	4.3 (1.9)	4.3 (1.8)	0.991	0.85 (0.48)	0.80 (0.51)	0.795
Pallidum	6.1 (4.1)	6.1 (4.1)	0.995	1.6 (1.0)	2.1 (1.1)	0.247	0.66 (0.43)	0.65 (0.44)	0.995
Cortical subplate	11.1 (5.3)	7.9 (6.1)	0.135	3.1 (1.4)	2.6 (1.8)	0.413	1.25 (0.60)	0.88 (0.68)	0.135
Thalamus, polymodal association cortex related	21.3 (8.9)	19.1 (12.7)	0.604	6.7 (2.5)	6.9 (3.2)	0.837	1.68 (0.71)	1.51 (1.00)	0.604
Thalamus, sensory-motor cortex related	10.6 (5.2)	10.7 (5.9)	0.991	3.7 (2.7)	4.9 (3.9)	0.361	1.59 (0.77)	1.6 (0.89)	0.991
Striatum, dorsal region	30.2 (15)	21.9 (11)	0.099	8.8 (2.0)	7.9 (1.8)	0.213	1.16 (0.57)	0.84 (0.42)	0.099
Striatum, ventral region	8.3 (4.9)	5.6 (3.4)	0.094	2.5 (0.9)	2.3 (1.2)	0.623	0.96 (0.57)	0.65 (0.39)	0.094
Other striatum regions	4.1 (2.9)	3.7 (2.6)	0.642	1.3 (1.0)	1.4 (0.7)	0.849	0.54 (0.37)	0.48 (0.34)	0.642
Olfactory areas	25.9 (16.9)	18.1 (10.4)	0.146	7.4 (2.9)	7.1 (2.9)	0.749	0.55 (0.36)	0.39 (0.22)	0.146
Agranular insular area	8.4 (3.9)	7.2 (4.3)	0.431	2.8 (1.0)	2.6 (1.3)	0.652	1.07 (0.49)	0.91 (0.55)	0.431
Auditory areas	8.4 (6.6)	4.9 (3.2)	0.089	2.2 (1.6)	1.8 (1.0)	0.403	1.45 (1.13)	0.85 (0.56)	0.089
Retrosplenial area	8.0 (6.0)	5.4 (3.6)	0.164	2.0 (1.3)	2.1 (1.5)	0.815	0.76 (0.57)	0.51 (0.34)	0.164
Somatosensory areas	33.5 (19.2)	28.7 (14.8)	0.453	9.3 (3.7)	10.6 (3.6)	0.320	1.01 (0.58)	0.86 (0.44)	0.453
Somatomotor areas	22.9 (14.4)	20.8 (11.7)	0.665	6.5 (3.3)	7.8 (2.7)	0.263	0.94 (0.59)	0.85 (0.48)	0.665
Visual areas	12.5 (7.8)	7.4 (6.2)	0.060	4.0 (3.0)	2.4 (1.8)	0.098	0.93 (0.58)	0.55 (0.46)	0.060
Orbital area	8.1 (6.0)	4.8 (4.3)	0.100	2.5 (2.3)	1.7 (1.5)	0.277	1.37 (1.01)	0.81 (0.73)	0.100
Other isocortex areas	20.9 (12.3)	15.7 (10.1)	0.224	5.7 (2.4)	5.9 (2.5)	0.870	0.95 (0.56)	0.72 (0.46)	0.224
Retrohippocampal area	8.8 (6.4)	6.8 (7.4)	0.450	2.4 (1.4)	2.2 (2.2)	0.777	0.48 (0.35)	0.37 (0.40)	0.450
Hippocampal region	28.3 (13.2)	23.7 (11.7)	0.332	9.7 (5.5)	8.6 (2.8)	0.507	1.19 (0.55)	1.00 (0.49)	0.332
Unaccounted microspheres	3.0 (2.6)	1.9 (1.6)	0.193	0.8 (0.6)	0.7 (0.5)	0.517			
Total, all regions combined	337.6 (153.6)	270.8 (117.3)	0.198	100	100		0.922 (0.408)	0.738 (0.319)	0.186

The total row for microsphere number and microsphere proportion are the sum of all regions within their respective columns. The total row for microsphere density uses the mean of all regions within its column. Data within a given column are means with SDs in parentheses.

10.1523/ENEURO.0216-21.2021.ed4 Extended Data 4 Detailed statistics comparing the distribution of microspheres across identified brain regions. Download Extended Data 4, ZIP file.

## Discussion

Experimental approaches that introduce microemboli into the brain, such as fluorescent microspheres, represent a promising preclinical model that mimics the global distribution of microinfarcts across the brain. We developed and validated an approach to semi-automatically localize all fluorescent microspheres from individual brains processed through serial block-face images or histology. By using this workflow, we showed that the anatomic location (e.g., somatomotor areas, hippocampal region, visual areas, etc.) of each microsphere within the brain could be accurately identified. To demonstrate the potential of incorporating this technique, we assessed whether transcardial perfusion at killing changed how microspheres clustered in the brain. While perfusion did not significantly change where microspheres were located, our data provide a comprehensive characterization of how microspheres are distributed throughout the entire mouse brain. Indeed, this is the first study to perform brain-wide localization of microemboli within the rodent brain and provides a high-throughput method for deriving regional microinfarct load within the brain.

We chose to employ QuickNII (Puchades et al., 2019) as it requires minimal user-guided manual transformations, does not introduce distortions in the original experimental images, and provides AMB coordinates for each experimental image to facilitate the registration of segmented objects (i.e., microspheres) to anatomic structures (Groeneboom et al., 2020). Through our block-face imaging workflow, an experimenter can localize all microspheres in the brain to anatomic regions defined by the AMB in less than 3 h (includes vibratome sectioning, image acquisition, and analysis). A significant advantage of registering block-face images instead of histologic images is that histology can introduce tissue folding or deformation, which increases the number of user-guided transformations necessary to register coronal histologic images to the AMB atlas and may decrease the final accuracy of registration. Compared with histologic methods, which require additional processing steps (staining with a structural marker, microscope imaging, etc.), the block-face imaging approach significantly decreased the time investment required to characterize the distribution of microspheres within the mouse brain (from days to hours). Likely because of increased tissue handling and tissue deformation, registering the location of microspheres from histologic sections also introduced sources of error. For example, when examining histologic images, 7.5% of microspheres were lost and the ability to localize microspheres to more defined anatomic regions (i.e., higher anatomic levels of the AMB) was reduced. Importantly, by using our block-face imaging workflow, experimenters can use a combination of slide-mounted or free-floating brain sections for additional histologic analysis to probe underlying mechanisms, without compromising the characterization of total microsphere number and location.

Our limited understanding of microinfarct distribution within the human brain or in animal models is in large part because of the laborious methods for globally characterizing microinfarcts in the brain. In animals, studies have typically sampled all microspheres in a limited number of animals (Silasi et al., 2015; Balbi et al., 2019) or a small subset of microspheres (0.03−0.15% of total injected) in each animal (Lam et al., 2010; van der Wijk et al., 2019, 2020). Here, we characterized the distribution of fluorescent microspheres in the largest group of mice to date. Similar to others that characterized microsphere distribution in mice (Silasi et al., 2015; Balbi et al., 2019), we found that ∼35% of microspheres are located in the isocortex and 5% are within fiber tracts. However, normalizing microsphere number to the size of the brain structure, revealed that microspheres are most densely located in the thalamus. In humans, small lacunar lesions in the thalamus independently predict cognitive decline with aging (Gold et al., 2005), potentially explaining why cognitive deficits have been observed with microemboli models (Rapp et al., 2008; Wang et al., 2012; Balbi et al., 2019). Microinfarcts are also wide-spread throughout the human brain and may also be more commonly located in cerebral cortex (Brundel et al., 2012). By using our localization workflow, researchers will be able to limit the potential for sampling bias while facilitating the ability to investigate the impact of microinfarct load and location on functional outcomes. For example, although it is reported that CCA injection of microspheres can result in behavioral impairments (Silasi et al., 2015; Balbi et al., 2019), the injury profile between mice is heterogenous. To better understand the relationship between microsphere-induced microocclusions and behavior, the total number and location of microspheres within the brain should be related to each animals’ behavioral data. Indeed, microinfarcts placed strategically within the brain can produce detectable behavioral impairments (Shih et al., 2013). To fully embrace the variability of microinfarct distribution within our model, automated behavioral paradigms (Silasi et al., 2018; Birtalan et al., 2020; Salameh et al., 2020) may be used to reveal the functional consequences of microocclusion load within sensorimotor or cognitive networks.

The majority of the studies using the fluorescent microsphere model have focused on the endogenous capacity of the brain endothelium to extravasate and clear microemboli, through the process termed angiophagy (Lam et al., 2010; van der Wijk et al., 2019, 2020). In fact, it is suggested that up to 80% of emboli are cleared from within the vessel lumen by day 7 postinjection and 100% are cleared by day 28 (Lam et al., 2010; van der Wijk et al., 2019, 2020). To date, with experimenters injecting upwards of 25,000 microspheres, only a small number of these microspheres are sampled (0.03−0.15%). Our analysis pipeline could facilitate the brain-wide localization of microemboli, allowing for a detailed regional analysis of microemboli extravasation frequency across brain regions.

To gain a better understanding of the behavioral impact of microinfarcts or the mechanisms associated with brain-wide microocclusions, it is essential that researchers begin to account for region-specific differences. Going forward, the developed block-face imaging workflow can facilitate the stratification of animals based on microocclusion load or location, to answer more complex questions regarding the impact of brain microinfarction on brain health.

## Synthesis

Reviewing Editor: Matthew Grubb, King's College London Decisions are customarily a result of the Reviewing Editor and the peer reviewers coming together and discussing their recommendations until a consensus is reached. When revisions are invited, a fact-based synthesis statement explaining their decision and outlining what is needed to prepare a revision will be listed below. The following reviewer(s) agreed to reveal their identity: Adam Tyson.

Both reviewers agree that this is a good paper that needs just a few minor revisions before being suitable for publication in eNeuro. Please make sure you address all of the reviewers' points in your resubmitted manuscript, and if at all possible please also include a working link to the software.

Reviewer 1:

This paper describes an automated platform for imaging and quantifying fluorescent micro-emboli throughout the mouse brain. The methods, which are the focus of this paper, are well described and I appreciate the multiple approaches used to validate their pipeline. Since there is increasing interest in using microspheres to model micro-emboli, I think this report will be useful to many labs. I do however, have some questions about the methods and results that should be addressed by the authors.

Questions/Comments:

1) Methods: please provide more detail on thresholding of signal. In some instances it was done manually and others with an automated but unspecified, at least in the methods, algorithm (Li, Huang, Otsu etc)? This is an especially important component because the images in Fig. 2A show that some bright microspheres were excluded after the threshold, suggesting it is either quite stringent based on fluorescence intensity or includes additional factors like blurring of signal. The manual threshold must have involved some very specific instructions to avoid counting these bright spots in the tissue.

2) Methods, what is the resolution of the imaging system, especially in the Z plane?

3) Just curious, how sensitive is the automated brain registration to slight asymmetries in brain sectioning, when one hemisphere is more posterior than the other?

4) Results, the ‘disagreement’ in results for the corpus callosum is quite high (25%), what factors account for this?

5) Results, regarding the statement on 267-268 that ‘majority microspheres were in isocortex, then thalamus and midbrain region", presumably this was not normalized to volume. Please make this explicitly clear since Table 3 shows no regional patterns when normalized to volume. Further to this point, the results states that when mice were collapsed into a single group, the highest density of microspheres was in the thalamus and the least was in certain fibre tracts. This is an important statement but no statistics or graphs (with variances) are presented to specifically support it. Please provide better support for this statement or remove it since the reader cannot easily see why it is justified.

6) Somewhere in the intro, methods or beginning of the results, the authors should clarify what parts of the vascular tree these microspheres are obstructing. Is it always in penetrating arterioles or distal ends of MCA/ACA (before becoming a penetrating arteriole)?

7) Since the occlusion of a deep penetrating arteriole might disrupt blood flow upstream, did the authors ever compute an analysis of clustering (nearest neighbour?) to determine if each microsphere is an independent event or perhaps a secondary consequence of a deeper obstruction?

8) Discussion: regarding the statement that ‘this is the first study to perform brain-wide localization of microemboli with the rodent brain...". Please note a recent study (PMID: 31903837) did exactly this but with smaller fluorescent microspheres (ones that don't induce stroke is the main distinction).

Reviewer 2:

In this study, the authors describe a straightforward method for imaging and analysis of fluorescent microspheres throughout the mouse brain. Their block-face approach compares favorably with ‘traditional’ slice histology and automated analysis (integrated with the existing QuickNII tool) works well. The paper is well written, and the approach is simple and well described, and I only have a few minor comments:

In the introduction, there are many more relevant imaging and analysis approaches that could be cited in addition to QuickNII. It would be useful to (briefly) outline other imaging approaches such as 3D whole-brain microscopy, e.g. light-sheet microscopy in cleared tissue or other block-face approaches such as serial section two-photon microscopy and FAST. There are also many existing tools for registration of 2D & 3D histology data (Wholebrain, ClearMap, brainreg, SHARP-TRACK, AMaSiNe etc.).

In Figure 2, the ‘Threshold selection’ and ‘Manual selection’ data points appear to be exactly overlapping (even at a pixel level in the ‘Merge’ panel). When the data was manually annotated, was this performed blind to the automated results? If so it is very unlikely that the microsphere positions would be detected in exactly the same place in both automated and manual methods. The exact approach of manual analysis should be outlined.

In Fig 4A, it would be helpful to have some kind of legend to match the colors to brain regions.

It would be helpful for the reader if the rationale behind the outlined approach (imaging and QuickNII integration) were justified. Is it easier, quicker, cheaper etc. than other methods?
