# Supplementary material for: Localizing Microemboli within the Rodent Brain through Block-Face Imaging and Atlas Registration
Source: eNeuro. 2021 Aug 2;8(4):ENEURO.0216-21.2021. doi: 10.1523/ENEURO.0216-21.2021 (PMC8342264; doi:10.1523/ENEURO.0216-21.2021)
Supplement: Extended Data 1 — Detailed description of the block-face imaging workflow and Python script for image acquisition. Download Extended Data 1, ZIP file. [file enu-eN-MNT-0216-21-s03.zip › Extended Data 1/BlockfaceImagingWorkflow.docx]

The following SOP outlines our blockface imaging workflow to localize fluorescent microspheres in the mouse brain.

1. **Vibratome Sectioning & Imaging**

- To capture images using the Pi camera use the script called “get_image.py”
- Press any key to take a picture
- Files will be named using the following convention: “month-day-year_hour minute second_b.jpg”
  - QuickNII only supports 24-bit PNG and JPG file formats (up to a resolution of 16 megapixels) 🡪 with current setup images do not exceed this size
- Once imaging is complete, create a root directory for each brain and save all files within the root directory and in a folder named “1-Raw Images”: e.g. Brain1/1-Raw Images

1. **Automatically align images using 'StackReg' plugin**

- Select option 1.1 in the Fiji/ImageJ script.
- This script first rotates images based on a user-drawn line down the brain’s midline.
- Next, the images are aligned using the “StackReg” plugin and the “Rigid Body” transformation
  - **IMPORTANT:** Before pressing “Ok”, choose the image that you wish every other image to be aligned to.
- Images are resaved in new “2- Aligned Images” folder.
- The coloured image and the green only channel are saved.

**Optional: Select brighten images to make internal brain structures more visible for anchoring with QuickNII**

- Select option 1.2 in the Fiji/ImageJ script
- Original images will not be modified a new folder called “Brightened” will be added
- Subsequent thresholding to detect microspheres will be conducted on original images without brightness enhancement

1. **Run FileBuilder**

- Use the images located in the “2- Aligned Images” folder (or “Brightened” folder if deemed necessary.
- **IMPORTANT:** save the .xml file from FileBuilder in the directory folder from which the .xml file is created (2- Aligned Images or Brightened folder). If saved elsewhere, QuickNII will not be able to anchor blockface image to atlas.

1. **Run QuickNII**
   - Click “Manage Data”🡪”Load”🡪open the .xml file produced from FileBuilder
   - Double click on an image.

- **To set an anchor, click “Store”.**
- Go to the last blockface image where the anterior commissure crosses. Make necessary changes to the cutting angle and adjust A/P location in atlas if necessary. Select “Store”.
- Anchor to the blockface image where the corpus callosum first crosses.
- Anchor to the atlas at an anterior and posterior hippocampus location.
- Anchor to anterior image containing frontal cortex and olfactory region.
- Lastly, visually verify that transformations appear accurate throughout your serial images. Amend as necessary.
- **IMPORTANT:** Once anchoring is complete, in “Manage Data” save updated .xml file (saved in 2- Aligned Images), JSON (saved in root directory), Propagation (saved in root directory), and Export Slices saved in “~Export Slices (from Quick NII)”

1. **Detect microspheres**

- Select option 4 in the Fiji/ImageJ script
- The user is prompted to choose whether to threshold from green or colour images (process validated using green channel to threshold microspheres)
- Thresholded images are saved in “3-Thresholded Images” folder

1. **Divide ROI of coronal image by hemisphere**

- Select option 5 in the Fiji/ImageJ script
- ROIs are saved in order to obtain data from each hemisphere independently (within 2-Aligned Images folder).
- The user is prompted to draw a square around the left hemisphere. Based on this, the ROIs for left and right hemispheres are produced.

1. **Consolidate true microsphere location and resize images for Nutil**

- Select option 6 in the Fiji/ImageJ script
- Determines the “true” microsphere depth by subtracting serial images and resizes the image based on atlas images obtained from QuickNII

1. **Run Nutil to localize microspheres**
2. **Determine the area of coronal images if you would like to ultimately obtain microsphere densities**

- Select option 8 in the Fiji/ImageJ script
