# Supplementary material for: Localizing Microemboli within the Rodent Brain through Block-Face Imaging and Atlas Registration
Source: eNeuro. 2021 Aug 2;8(4):ENEURO.0216-21.2021. doi: 10.1523/ENEURO.0216-21.2021 (PMC8342264; doi:10.1523/ENEURO.0216-21.2021)

**Extended Data 4:** Detailed statistics comparing the distribution of microspheres across identified brain regions.

A Ryan-Einot-Gabriel-Welsh F test was used to determine which brain regions had similar densities of microspheres. Nine homogenous subsets were identified and the results are presented below. Briefly, the thalamus had the largest densities of microspheres and was determined to be its own homogenous subset.


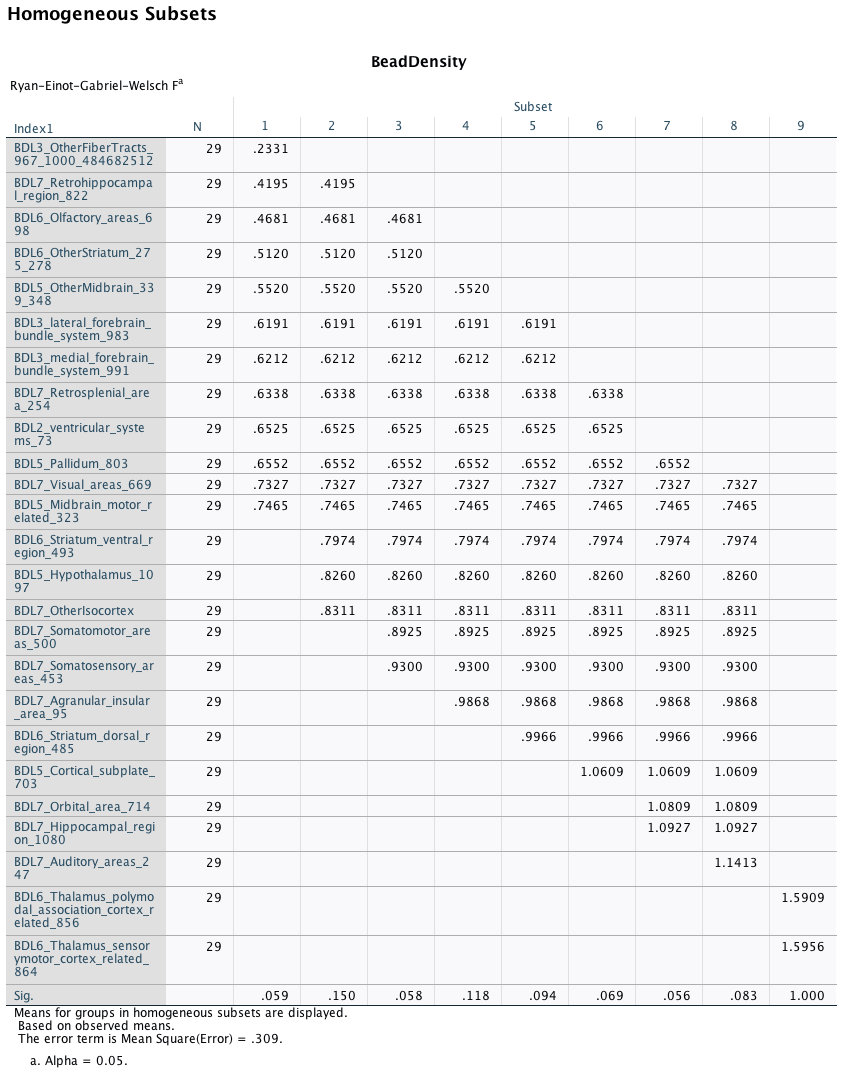

Supplement: Extended Data 4 — Detailed statistics comparing the distribution of microspheres across identified brain regions. Download Extended Data 4, ZIP file. [file enu-eN-MNT-0216-21-s02.zip › Extended Data 4.docx]
